# Molecular pharming in maize endosperm: A proof of concept with recombinant SARS‐CoV‐2 antigen production

**DOI:** 10.1111/pbi.70209

**Published:** 2025-06-15

**Authors:** Yuxin Tai, Shujun Meng, Yiteng Zhang, Jianfeng Weng, Xinhai Li, Qingyu Wu

**Affiliations:** ^1^ State Key Laboratory of Efficient Utilization of Arable Land in China, Institute of Agricultural Resources and Regional Planning Chinese Academy of Agricultural Sciences Beijing China; ^2^ Institute of Crop Sciences Chinese Academy of Agricultural Sciences Beijing China; ^3^ Institute of Crop Cultivation and Farming Heilongjiang Academy of Agricultural Sciences, Heilongjiang Academy of Agricultural Sciences Harbin China; ^4^ National Nanfan Research Institute (Sanya) Chinese Academy of Agricultural Sciences Sanya China

**Keywords:** SARS‐CoV‐2, Spike, RBD, maize, molecular pharming

Molecular pharming, which leverages plants to produce therapeutic proteins, is a cost‐effective, scalable and environmentally friendly method for recombinant protein production. It primarily involves expressing therapeutic proteins, such as antigens, in plants and subsequently purifying these proteins for pharmaceutical applications (Fausther‐Bovendo and Kobinger, 2021). Early pioneers identified several key advantages of plants as molecular pharming vessels, including their low cost, scalability, safety and environmental sustainability (Zhu *et al*., 2022). A variety of recombinant proteins have been successfully expressed in the transgenic lines of crops (Saba‐Mayoral *et al*., 2023). Among these crops, maize endosperm is particularly well‐suited as a bioreactor for producing and storing pharmaceutical proteins or oral vaccines for the following reasons: (1) it is structurally and compositionally stable for the storage of recombinant proteins; (2) the protein synthesis and modification machinery of endosperm cells can produce complex structural proteins with high activity; (3) the process of recombinant protein extraction and purification is easy; and (4) the production cost is low, since maize is a high‐yielding and the most widely cultivated cereal crop (Ramessar *et al*., 2008).

As a proof‐of‐concept demonstration of molecular pharming in maize endosperm, we report producing the proteins of the severe acute respiratory syndrome coronavirus 2 (SARS‐CoV‐2), the causal agent of the coronavirus 2019 (COVID‐19) pandemic, in this tissue. SARS‐CoV‐2 is an RNA virus encoding four structural proteins: spike (S), envelope (E), membrane (M) and nucleocapsid (N) (Masters, 2006). The S protein contains a furin cleavage site (RRAR) and it is a trimeric fusion protein, with each monomer comprising two subunits: S1 (for receptor attachment) and S2 (for membrane fusion) (Millet and Whittaker, 2015). The receptor‐binding domain (RBD) of S1 interacts with human angiotensin‐converting enzyme 2 (ACE2), a critical step for host cell entry (Wrapp *et al*., 2020). Consequently, the S protein, and especially its RBD, is the primary target of most SARS‐CoV‐2 vaccines.

We first tried to produce RBD in the maize endosperm. To ensure a strong and specific endosperm expression, we used the maize 19 kDa α‐zein promoter to drive its expression, as it has been shown to be effective in producing recombinant proteins in kernels. To ensure the proper modification of RBD, we added an endoplasmic reticulum (ER) signal peptide at its N‐terminus. To facilitate RBD purification and detection, we added the 2×StrepII‐3×FLAG tag at its C‐terminus and created a TEV cleavage site between RBD and the tag to enable tag‐free protein purification (Gloeckner *et al*., 2009) (Data S1). To maximize the expression of the RBD protein, we synthesized a maize codon‐optimized RBD coding sequence (Data S2) and introduced it into the pCAM3300 vector (Figures 1a and S1).

**Figure 1:**
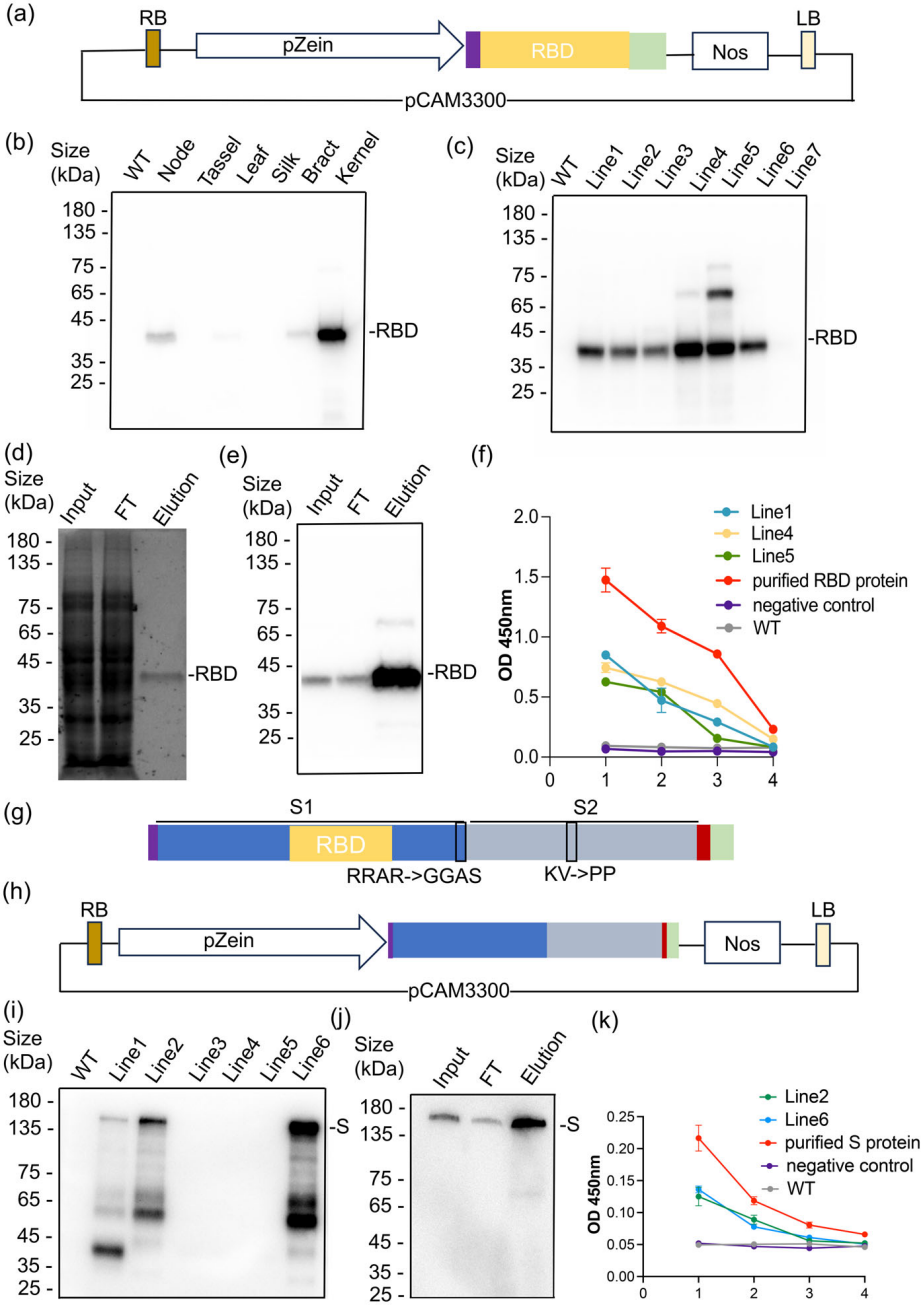
Expression and purification of RBD and S proteins produced from maize kernels and the binding ability to ACE2. (a) Vector map of the *pZein::RBD‐2×StrepII‐3×FLAG* construct. Purple box: signal peptide; green box: 2×StrepII‐3×FLAG Tag; RB: right border of T‐DNA; Nos: terminator; LB: left border of T‐DNA. (b) Western blot analysis from various tissues pooled from seven independent transgenic lines using an anti‐FLAG antibody. (c) Detection of the protein extracted from kernels using an anti‐FLAG antibody. Lines 1–7 represent different transgenic maize lines. (d) SDS‐PAGE analysis of purified RBD protein. Input: total protein; FT: proteins that did not bind to the purification beads; Elution: proteins that successfully bound to the beads. (e) Western blot analysis of the purified RBD protein using an anti‐FLAG antibody. (f) ELISA to determine the ability of crude extracts containing RBD to bind ACE2. The graph depicts binding efficiency, represented by absorbance on the y‐axis, across a range of dilutions on the x‐axis. Line 1, Line 4 and Line 5 represent three independent transgenic lines. Proteins were extracted three times (biological replicates) and each sample was measured three times (technical replicates). Data are means of all replicates ± SE (*n* = 9). (g) Schematic representation of the maize codon‐optimized recombinant S protein. Red box: fibritin trimerization motif. (h) Vector map of the *pZein::S*‐*2×StrepII‐3×FLAG* construct. (i) Detection of the maize codon‐optimized S protein expressed in transgenic plants. Lines 1–6 represent different transgenic lines. (j) Western blot analysis of purified maize codon‐optimized S protein. (k) Binding activity of the plant‐produced S protein with ACE2 was analysed by ELISA; Line 2 and Line 6 represent different transgenic lines. Data are means of all replicates ± SE (*n* = 9).

After sequence verification, the *pZein*::*RBD‐2×StrepII‐3×Flag* vector was introduced into *Agrobacterium tumefaciens*, which was then transformed into maize. Next, we investigated the expression pattern of RBD in different tissues, including node, tassel, leaf, silk, bract, and kernel. Western blot analysis revealed that RBD was predominantly expressed in kernels, exhibited weaker expression in node and bract tissues and was not detected in tassel, leaf or silk tissues (Figure 1b). A total of seven different transgenic lines were recovered, and six of these lines successfully expressed RBD (Figure 1c).

Next, we examined whether the RBD protein could be purified from the kernels. Using Strep‐Tactin XT magnetic beads to purify samples derived from maize kernels at 32 days post‐pollination, we recovered an average of 31.36 μg of the recombinant RBD protein per gram of fresh tissue, indicating that the system used for protein expression and purification was efficient. The sodium dodecyl sulfate‐polyacrylamide gel electrophoresis (SDS‐PAGE) (Figure 1d) and western blot (Figure 1e) indicated that the molecular weight of the RBD protein was approximately 38 kDa, as expected.

To test whether the RBD protein purified from the maize endosperm was bioactive and could bind to ACE2, we performed enzyme‐linked immunosorbent assay (ELISA) using the purified RBD protein and maize kernel extract, along with bovine serum albumin (BSA) and protein extracts from non‐transgenic kernels as negative controls. We found that the plant‐produced RBD protein displayed significantly higher signal than the negative control and wild‐type proteins. In addition, the signal decreased with the reduction in RBD protein concentration, confirming a specific and concentration‐dependent interaction between RBD and ACE2 (Figure 1f). These results confirm the structural integrity and *in vitro* binding activity of the maize endosperm‐produced RBD protein, indicating that maize kernels are suitable for developing scalable production platforms.

Using a similar strategy as described above, we expressed and purified the ectodomain of the SARS‐CoV‐2 S protein. To obtain the uncleaved full‐length protein, the S1/S2 furin cleavage site (RRAR) was mutated to GSAS (Gobeil *et al*., 2021). Additionally, the SARS‐CoV‐2 S protein was modified by deleting the C‐terminal transmembrane domain (to achieve efficient expression), introducing two proline substitutions (to stabilize the S2 fusion machinery), and incorporating a fibritin trimerization motif (to ensure proper trimer formation) (Wrapp *et al*., 2020) (Figure 1g) (Data S3).

Gene encoding the maize codon‐optimized S protein sequence (Data S4) was cloned into a vector under the control of the *pZein* promoter to generate a fusion with the 2×StrepII‐3×FLAG tag at its C‐terminus (Figure 1h and S2). Following sequence verification, the construct was introduced into maize via *Agrobacterium*‐mediated transformation. Six independent transgenic lines were recovered, three of which expressed the S protein, as confirmed by western blot analysis using anti‐FLAG antibody (Figure 1i). Among the six transgenic lines, Line 6 showed the highest protein accumulation and was selected for further analysis.

The recombinant S protein was purified from 15 g of ground maize kernels using the previously described method. Western blot analysis of the purified protein showed enrichment in the immunoprecipitation (IP) lane, with a major band observed at ~146 kDa (Figure 1j). These results confirm that the codon‐optimized S protein can be successfully expressed in maize kernels and efficiently purified. Similar to RBD, the ELISA assay showed that the plant‐produced S protein was also bioactive to ACE2 (Figure 1k).

Glycosylation is crucial for maintaining the structural integrity and biological activity of RBD and S proteins. To facilitate proper glycosylation, a signal peptide was fused to the N‐terminal region of the recombinant proteins, directing their transport from the ER to the Golgi apparatus. Although glycosylation patterns in maize endosperm may differ from those observed in animal systems, our ELISA results demonstrate that maize endosperm‐produced RBD and S proteins retain their ACE2‐binding capacity (Zhu *et al*., 2022). This confirms that essential functional glycosylation patterns are effectively preserved in our plant‐based expression system.

The maize endosperm‐produced RBD and S proteins have multiple practical applications that extend beyond proof‐of‐concept, particularly given their confirmed ability to bind human ACE2 in ELISA assays (Figure 1f,k). First, they can serve as antigenic components in vaccines against SARS‐CoV‐2. Plant‐based vaccines are highly scalable, safe and suitable for both injectable and edible booster strategies in prime‐boost immunization regimens. Second, these proteins can be used in diagnostic applications, including ELISA and lateral flow assays, to detect and quantify antibodies in patient sera. Third, these proteins can act as a bait in high‐throughput screening platforms for neutralizing antibodies, facilitating the development of therapeutic antibodies and the monitoring of host immune responses. Together, these use cases demonstrate the translational potential of plant‐produced SARS‐CoV‐2 proteins and support the feasibility of maize endosperm as a large‐scale biomanufacturing platform.

## Conflict of interest

The authors declare no competing interest.

## Author contributions

Y.T., S.M. and Y.Z. performed the experiments. J.W., X.L. and Q.W. wrote and revised the manuscript. All authors contributed to the article and approved the submitted version.

## Supporting information


**Figure S1** Vector map of the *pZein*::*RBD*‐*2×StrepII‐3×FLAG* in pCAM3300.
**Figure S2** Vector map of the *pZein*:: *S*‐*2×StrepII‐3×FLAG* in  pCAM3300.
**Data S1** Protein sequence of RBD.
**Data S2** Nucleotide sequence of codon‐optimized RBD.
**Data S3** Protein sequence of S.
**Data S4** Nucleotide sequence of codon‐optimized S.

## Data Availability

The data that supports the findings of this study are available in the supplementary material of this article.
