# Supplementary material for: Molecular pharming in maize endosperm: A proof of concept with recombinant SARS‐CoV‐2 antigen production
Source: Plant Biotechnol J. 2025 Jun 15;23(9):3838–40. doi: 10.1111/pbi.70209 (PMC12392926; doi:10.1111/pbi.70209)
Supplement: Supplementary file 1 — Figure S1 Vector map of the pZein::RBD‐2×StrepII‐3×FLAG in pCAM3300. Figure S2 Vector map of the pZein:: S‐2×StrepII‐3×FLAG in pCAM3300. Data S1 Protein sequence of RBD. Data S2 Nucleotide sequence of codon‐optimized RBD. Data S3 Protein sequence of S. Data S4 Nucleotide sequence of codon‐optimized S. [file PBI-23-3838-s001.docx]

**Supporting information:**

**
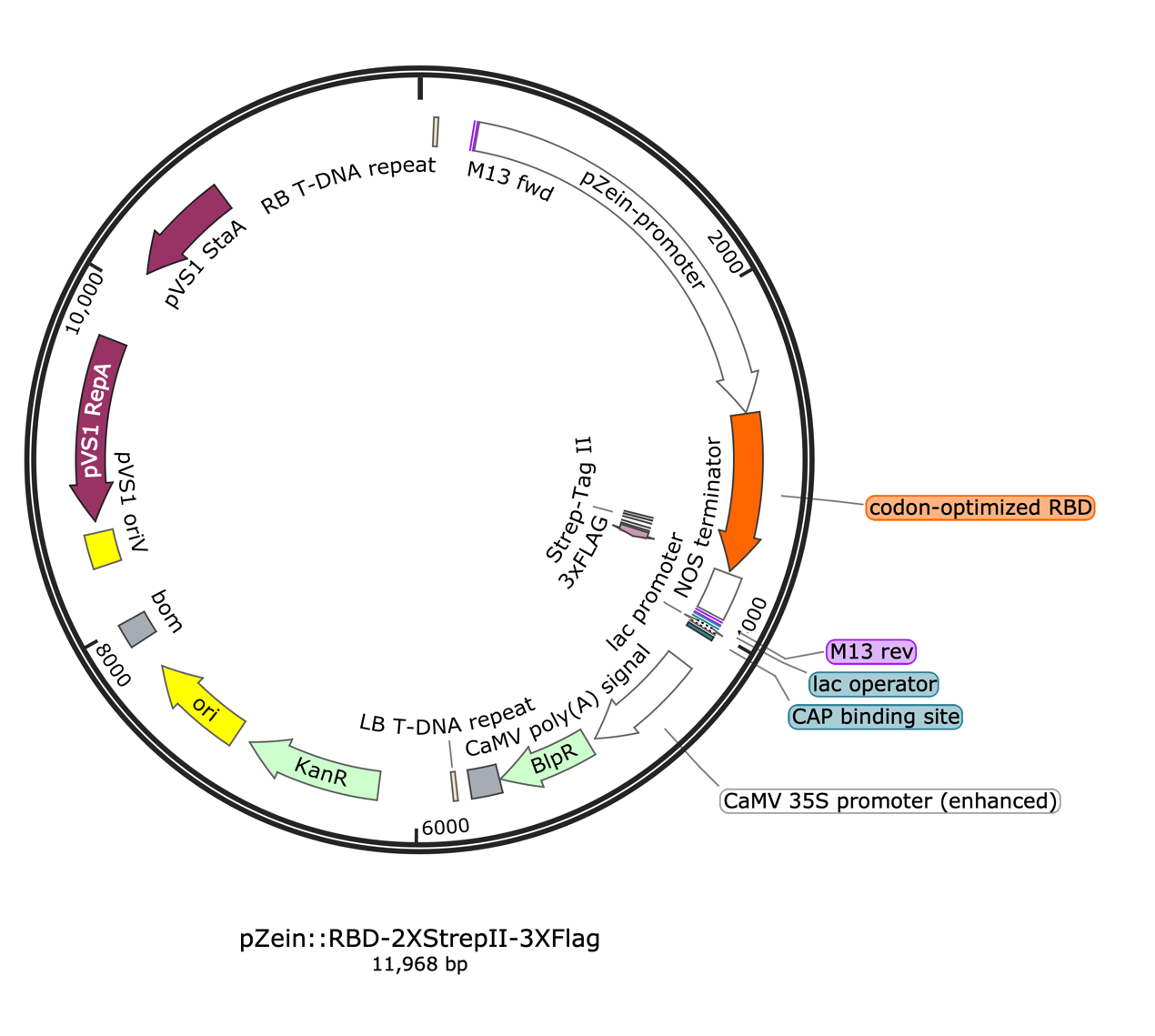
**

**Figure S1：**Vector map of the *pZein::RBD*-*2×StrepII-3×FLAG* in pCAM3300*.*


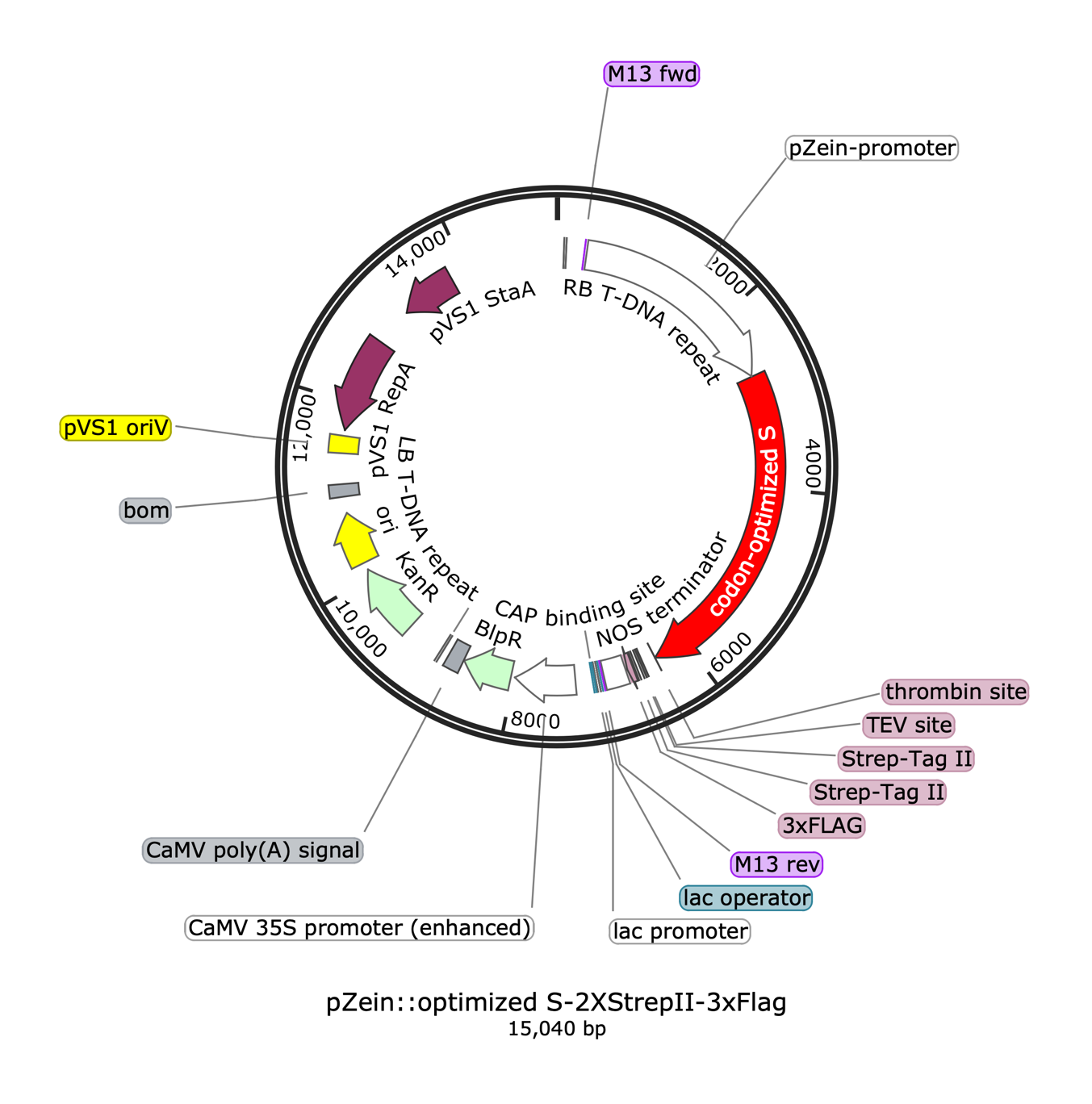


**Figure S2**: Vector map of the *pZein:: S*-*2×StrepII-3×FLAG* in pCAM3300*.*

**Data S1** Protein sequence of RBD.

MAAKIFCLLMLLGLSASAATARVQPTESIVRFPNITNLCPFGEVFNATRFASVYAWNRKRISNCVADYSVLYNSASFSTFKCYGVSPTKLNDLCFTNVYADSFVIRGDEVRQIAPGQTGKIADYNYKLPDDFTGCVIAWNSNNLDSKVGGNYNYLYRLFRKSNLKPFERDISTEIYQAGSTPCNGVEGFNCYFPLQSYGFQPTNGVGYQPYRVVVLSFELLHAPATVCGPKKSTNLVKNKCVNFENLYFQGSWSHPQFEKGGGSGGGSGGGSWSHPQFEKGASGEDYKDHDGDYKDHDIDYKDDDDK

Yellow: ER signal peptide

Green: RBD protein

Blue: TEV cleavage site

Purple: 2×StrepII-3×FLAG tag

**Data S2** Nucleotide sequence of codon-optimized RBD.

ATGGCCGCTAAGATCTTCTGCCTGCTGATGCTGCTGGGCCTGTCCGCCTCCGCCGCTACTGCTAGGGTGCAGCCGACCGAGTCCATCGTGAGGTTCCCGAACATCACCAACCTGTGCCCGTTCGGCGAGGTGTTCAATGCCACCAGGTTCGCTTCCGTCTACGCCTGGAATCGGAAGAGGATCTCTAACTGCGTGGCGGACTACTCTGTGCTCTACAACTCCGCCTCCTTCTCCACCTTCAAGTGCTACGGCGTGTCCCCGACCAAGCTGAACGATCTGTGCTTCACCAACGTCTACGCCGACTCCTTCGTGATTCGCGGCGACGAGGTCCGCCAGATCGCCCCAGGCCAGACCGGCAAGATTGCCGACTACAACTACAAGCTGCCCGACGATTTCACCGGCTGCGTGATTGCCTGGAACTCCAACAACCTGGACTCCAAGGTGGGCGGCAACTACAACTACCTGTACAGGCTGTTCAGGAAGAGCAACCTCAAGCCGTTCGAGAGGGACATCAGCACGGAGATCTACCAGGCCGGCTCCACCCCATGCAACGGCGTGGAGGGCTTCAACTGCTACTTCCCCCTCCAGTCCTACGGCTTCCAGCCAACCAACGGCGTGGGCTACCAGCCATACCGCGTGGTCGTGCTCTCCTTCGAGCTGCTCCACGCCCCGGCTACGGTGTGCGGCCCAAAGAAGTCCACCAACCTGGTGAAGAACAAGTGCGTGAACTTCGAGAACCTGTACTTCCAGGGCTCCTGGTCCCACCCGCAGTTCGAGAAGGGCGGCGGCTCAGGCGGCGGCTCCGGCGGCGGCAGCTGGTCCCACCCACAGTTCGAGAAGGGCGCCTCCGGCGAGGATTACAAGGACCACGACGGCGACTACAAGGACCATGACATCGACTACAAGGATGATGACGACAAGTAA

**Data S3** Protein sequence of S.

MAAKIFCLLMLLGLSASAATAVNLTTRTQLPPAYTNSFTRGVYYPDKVFRSSVLHSTQDLFLPFFSNVTWFHAIHVSGTNGTKRFDNPVLPFNDGVYFASTEKSNIIRGWIFGTTLDSKTQSLLIVNNATNVVIKVCEFQFCNDPFLGVYYHKNNKSWMESEFRVYSSANNCTFEYVSQPFLMDLEGKQGNFKNLREFVFKNIDGYFKIYSKHTPINLVRDLPQGFSALEPLVDLPIGINITRFQTLLALHRSYLTPGDSSSGWTAGAAAYYVGYLQPRTFLLKYNENGTITDAVDCALDPLSETKCTLKSFTVEKGIYQTSNFRVQPTESIVRFPNITNLCPFGEVFNATRFASVYAWNRKRISNCVADYSVLYNSASFSTFKCYGVSPTKLNDLCFTNVYADSFVIRGDEVRQIAPGQTGKIADYNYKLPDDFTGCVIAWNSNNLDSKVGGNYNYLYRLFRKSNLKPFERDISTEIYQAGSTPCNGVEGFNCYFPLQSYGFQPTNGVGYQPYRVVVLSFELLHAPATVCGPKKSTNLVKNKCVNFNFNGLTGTGVLTESNKKFLPFQQFGRDIADTTDAVRDPQTLEILDITPCSFGGVSVITPGTNTSNQVAVLYQDVNCTEVPVAIHADQLTPTWRVYSTGSNVFQTRAGCLIGAEHVNNSYECDIPIGAGICASYQTQTNSP**GSAS**SVASQSIIAYTMSLGAENSVAYSNNSIAIPTNFTISVTTEILPVSMTKTSVDCTMYICGDSTECSNLLLQYGSFCTQLNRALTGIAVEQDKNTQEVFAQVKQIYKTPPIKDFGGFNFSQILPDPSKPSKRSFIEDLLFNKVTLADAGFIKQYGDCLGDIAARDLICAQKFNGLTVLPPLLTDEMIAQYTSALLAGTITSGWTFGAGAALQIPFAMQMAYRFNGIGVTQNVLYENQKLIANQFNSAIGKIQDSLSSTASALGKLQDVVNQNAQALNTLVKQLSSNFGAIS

SVLNDILSRLD**PP**EAEVQIDRLITGRLQSLQTYVTQQLIRAAEIRASANLAATKMSECVLGQSKRVDFCGKGYHLMSFPQSAPHGVVFLHVTYVPAQEKNFTTAPAICHDGKAHFPREGVFVSNGTHWFVTQRNFYEPQIITTDNTFVSGNCDVVIGIVNNTVYDPLQPELDSFKEELDKYFKNHTSPDVDLGDISGINASVVNIQKEIDRLNEVAKNLNESLIDLQELGKYEQYIKWPLVPRGSGSGSGSGSGYIPEAPRDGQCYVRCDGEWVLLSTFLGGSGSGSGENLYFQGSWSHPQFEKGGGSGGGSGGGSWSHPQFEKGASGEDYKDHDGDYKDHDIDYKDDDDK

Yellow: ER signal peptide

Gray: S1 subunit

Green: S2 subunit

Red: fibritin trimerization motif

Blue: TEV cleavage site

Purple: 2×StrepII-3×FLAG tag

Red characters indicate the amino acid substitutions

**Data S4** Nucleotide sequence of codon-optimized S.

ATGGCCGCTAAGATCTTCTGCCTGCTGATGCTGCTGGGCCTGTCCGCCTCCGCCGCTACTGCTGTGAACCTGACCACCAGGACCCAGCTGCCGCCGGCTTACACCAACTCCTTCACCAGGGGCGTGTACTACCCCGATAAGGTGTTCCGCTCCTCCGTGCTGCATTCCACCCAGGACCTGTTCCTGCCGTTCTTCTCCAACGTCACCTGGTTCCATGCCATCCACGTCTCCGGCACCAATGGCACCAAGCGCTTCGACAATCCGGTCCTCCCCTTCAACGACGGCGTGTACTTCGCCTCCACCGAGAAGTCCAACATCATCCGCGGCTGGATCTTCGGCACCACCCTCGACTCCAAGACCCAGTCCCTGCTTATCGTCAACAATGCCACCAACGTGGTGATCAAGGTGTGCGAGTTCCAGTTCTGCAATGACCCGTTCCTGGGCGTGTACTACCACAAGAACAACAAGTCCTGGATGGAGTCCGAGTTCAGGGTTTACTCCTCTGCCAACAATTGCACCTTCGAGTACGTGTCCCAGCCCTTCCTGATGGACCTGGAGGGCAAGCAGGGCAATTTCAAGAACCTGCGCGAGTTCGTGTTCAAGAACATCGATGGCTACTTCAAGATCTACTCCAAGCACACACCGATCAATCTCGTCCGCGACCTGCCGCAGGGCTTCTCCGCTCTGGAGCCCCTGGTCGACCTCCCAATCGGCATCAACATCACCCGCTTCCAGACCCTGCTGGCCCTCCACCGCTCCTACCTGACCCCAGGCGACTCCTCCTCCGGCTGGACCGCTGGCGCTGCTGCTTACTACGTGGGCTACCTGCAGCCGCGCACCTTCCTCCTCAAGTACAACGAGAACGGCACCATCACCGACGCCGTGGACTGCGCCCTCGACCCACTGAGCGAGACCAAGTGCACCCTGAAGTCCTTCACCGTGGAGAAGGGCATCTACCAGACCTCCAACTTCAGGGTGCAGCCGACCGAGAGCATTGTGAGGTTCCCGAACATCACGAACCTGTGCCCGTTCGGCGAGGTGTTCAACGCCACCCGCTTCGCCTCCGTGTACGCCTGGAACAGGAAGAGGATCTCCAACTGCGTGGCCGACTACTCTGTGCTCTACAACTCCGCCTCCTTCTCCACCTTCAAGTGCTACGGCGTGTCCCCGACCAAGCTGAACGATCTGTGCTTCACCAACGTCTACGCCGACTCCTTCGTGATCCGCGGCGACGAGGTCCGCCAGATCGCCCCAGGCCAGACCGGCAAGATCGCCGATTACAACTACAAGCTGCCGGACGACTTCACCGGCTGCGTGATTGCCTGGAACTCCAACAACCTGGACTCCAAGGTGGGCGGCAACTACAACTACCTGTACAGGCTTTTCCGCAAGAGCAACCTGAAGCCGTTCGAGAGGGACATCAGCACGGAGATCTACCAGGCCGGCTCTACCCCATGCAACGGCGTGGAGGGCTTCAACTGCTACTTCCCGCTGCAGTCCTACGGCTTCCAGCCAACCAACGGCGTCGGCTACCAGCCGTACAGGGTGGTCGTGCTCTCCTTCGAGCTGCTCCACGCCCCGGCGACCGTGTGCGGCCCAAAGAAGTCCACCAACCTTGTGAAGAACAAGTGCGTGAACTTCAACTTCAACGGCCTTACCGGCACCGGCGTCCTTACCGAGAGCAACAAGAAGTTCCTCCCGTTCCAGCAGTTCGGCAGGGACATCGCCGACACCACCGACGCCGTCAGGGACCCACAGACCCTGGAGATTCTCGACATCACCCCGTGCTCCTTCGGCGGCGTGTCCGTGATCACCCCAGGCACCAACACCAGCAACCAGGTCGCCGTGCTGTACCAGGACGTGAACTGCACCGAGGTGCCTGTGGCCATCCACGCCGACCAGCTGACCCCCACCTGGAGGGTCTACTCCACCGGCTCCAACGTGTTCCAGACCAGGGCCGGCTGCCTGATCGGCGCTGAGCACGTGAACAACTCCTACGAGTGCGACATCCCGATTGGCGCCGGCATCTGCGCCTCCTACCAGACCCAGACAAACTCCCCAGGTTCCGCCTCCTCCGTGGCCTCCCAGTCAATCATTGCCTACACCATGTCCCTGGGCGCCGAGAACTCCGTGGCGTACTCAAACAACTCCATCGCCATCCCAACCAACTTCACTATCTCCGTGACCACCGAGATTCTTCCCGTCTCCATGACCAAGACCTCCGTGGACTGCACGATGTACATCTGCGGCGACTCCACCGAGTGCTCCAACCTGCTGCTCCAGTACGGCTCCTTCTGCACCCAGCTGAACCGCGCCCTCACCGGCATCGCCGTGGAGCAGGACAAGAACACCCAGGAGGTGTTCGCCCAGGTGAAGCAGATCTACAAGACCCCGCCGATCAAGGATTTCGGCGGCTTCAACTTCAGCCAGATCCTCCCGGACCCCTCCAAGCCAAGCAAGAGGTCCTTCATCGAGGATCTTCTGTTCAATAAGGTCACCCTCGCCGACGCCGGCTTCATCAAGCAGTACGGCGACTGCCTCGGCGACATCGCCGCCAGGGACCTCATCTGCGCCCAGAAGTTCAACGGCCTGACCGTCCTGCCGCCCCTGCTGACTGACGAGATGATCGCGCAGTACACCTCCGCCCTGCTGGCCGGCACGATCACCTCCGGCTGGACTTTCGGCGCCGGCGCTGCTCTCCAGATTCCCTTCGCCATGCAGATGGCCTACCGCTTCAACGGCATCGGCGTCACCCAGAACGTTCTCTACGAGAACCAGAAGCTGATCGCGAACCAGTTCAACTCCGCGATCGGCAAGATCCAGGACTCCCTGTCCTCCACCGCCTCCGCCCTCGGCAAGCTGCAGGACGTGGTGAACCAGAACGCCCAGGCCCTCAACACCCTGGTGAAGCAGCTCAGCTCCAACTTCGGCGCCATCTCCTCTGTGCTGAACGACATCCTGTCCCGCCTCGATCCGCCCGAGGCCGAGGTTCAGATCGACCGCCTCATCACCGGCAGGCTCCAGTCCCTCCAGACCTACGTGACCCAGCAGCTGATCCGCGCCGCCGAGATCCGCGCTTCTGCCAACCTTGCCGCCACCAAGATGTCCGAGTGCGTGCTCGGCCAGTCCAAGAGGGTTGACTTCTGCGGCAAGGGCTACCACCTCATGTCCTTCCCACAGTCCGCGCCGCACGGCGTGGTTTTCCTCCATGTGACCTACGTGCCGGCGCAGGAGAAGAACTTCACCACAGCGCCGGCCATCTGCCACGACGGCAAGGCTCACTTCCCCAGGGAGGGCGTGTTCGTTTCCAACGGCACCCACTGGTTCGTGACCCAGCGCAATTTCTACGAGCCGCAGATCATCACCACCGACAACACCTTCGTGTCCGGCAACTGCGACGTGGTTATCGGCATTGTCAACAATACCGTGTACGACCCCCTGCAGCCGGAGCTTGACTCCTTCAAGGAGGAGCTTGACAAGTACTTCAAGAACCACACCTCGCCGGACGTGGACCTGGGCGATATTTCTGGCATTAACGCCTCCGTTGTGAACATCCAGAAGGAGATCGACAGGCTTAACGAGGTGGCCAAGAACCTGAACGAGTCCCTGATCGACCTTCAGGAGCTGGGCAAGTACGAGCAGTACATTAAGTGGCCGCTGGTGCCGAGGGGCTCCGGCTCCGGCAGCGGCTCCGGCTCTGGCTACATCCCGGAGGCCCCGAGGGACGGCCAGTGCTACGTGAGGTGCGACGGCGAGTGGGTGCTGCTGTCTACGTTCCTGGGCGGCTCCGGCTCAGGCAGCGGCGAGAATCTGTACTTCCAGGGCTCCTGGTCCCACCCGCAGTTCGAGAAGGGCGGCGGCTCCGGCGGCGGCAGCGGCGGCGGCTCTTGGTCCCACCCACAGTTCGAGAAGGGCGCGAGCGGCGAGGACTACAAGGACCACGACGGCGACTACAAGGATCACGACATCGATTACAAGGACGATGATGACAAGTAA

**Experimental Procedures**

**Vector construction and maize transformation**

The maize codon-optimized sequences of the RBD protein and S protein ectodomain, with amino acid substitutions described previously (Wrapp *et al*., 2020), were synthesized. The sequence of the ectodomain of the S protein also included the fibritin trimerization motif and a C-terminal tag (2×StrepII-3×FLAG) for purification. The synthesized sequences were cloned into the pCAM3300 vector (Supplementary information). To achieve endosperm-specific expression, the ubiquitin promoter in the pCAM3300 vector was replaced with the maize 19-kDa α-Zein promoter. The final constructs were validated and introduced into *Agrobacterium tumefaciens*, which was subsequently transformed into the maize inbred line KN5585.

**Protein extraction and purification**

To prepare maize protein extract, kernels were harvested at 30 days after pollination and ground into a fine powder under liquid nitrogen. Protein extraction buffer (100 mM Tris-HCl [pH 8.0], 150 mM NaCl, 0.1% SDS, 1 mM EDTA, 5% glycerol, 1% TritonX-100, and protease inhibitor cocktail [Cat # 1183617001, Millipore Sigma]) was added to the powdered maize kernels (2 μl of protein extraction buffer for every gram of tissue). The sample was gently mixed and rotated at 4°C for 30 min. Samples were then centrifuged at 12,000 × *g* for 15 min at 4°C. The supernatant was collected and filtered through a 0.45-μm filter to obtain the total protein extract. Then, the supernatant was mixed with MagStrep Strep-Tactin XT beads (IBA-lifesciences) for 30 min at 4°C, and the protein was affinity purified according to the manufacturer’s instructions.

Proteins were separated by SDS-PAGE using 10% polyacrylamide gels in SDS running buffer (25 mM Tris, 250 mM glycine, 0.1% SDS) for 35 min at 200 V. To perform western blot analysis, proteins were transferred on to polyvinylidene fluoride (PVDF) membranes (Millipore) via semi-dry transfer at 12 V for 25 min. The membranes were blocked with TBST (TBS with 0.05% Tween-20) containing 5% skim milk for 2 h at room temperature, incubated with anti-FLAG M2 antibody (1:5000 dilution, Cat# F1804, Millipore Sigma) for 1.5 h at room temperature, and then washed five times with TBST for 10 min each time. Then, the membranes were incubated with Horse Radish Peroxidase (HRP)-conjugated secondary antibody (1:10000 dilution, Cat# 31458, ThermoFisher) for 1 h at room temperature and then subjected to another five 10-min washes with TBST. Immunoblots were developed using Amersham ECL detection reagents (Report medicine RPN2105, Cytiva) and imaged with a Fujifilm LAS-3000 imager.

**ELISA**

Coaster 96-well plates (Corning) were coated with 10 μg/ml of ACE2 (Cat#287935, Abcam) in bicarbonate buffer (pH = 9.6) and incubated overnight at 4ºC overnight. After incubation, the coating buffer was discarded, and the plates were washed three times with PBST (PBS supplemented with 0.05% Tween-20). The wells were blocked with 1% BSA in PBST for 1 h at 37°C to prevent nonspecific binding. Following blocking, the plates were incubated with the plant-produced RBD protein for 2 h at 37°C. At this step, 1% BSA in PBST was used as a negative control. After three washes with PBST, the plates were incubated with anti-FLAG antibody (1:1000 dilution) for 2 h at room temperature. The plates were washed again and incubated with Horse Radish Peroxidase (HRP)-conjugated secondary antibody (1:5000 dilution, Cat#AP308P, Millipore Sigma) for 1 h at 37°C. After the final washing steps, the fluorescence signal was developed using the TMB substrate (Cat# G7431, Promega), and absorbance was measured at 450 nm using a microplate reader.
